# Machine Learning Based Prediction of Insufficient Herbage Allowance with Automated Feeding Behaviour and Activity Data

**DOI:** 10.3390/s19204479

**Published:** 2019-10-16

**Authors:** Abu Zar Shafiullah, Jessica Werner, Emer Kennedy, Lorenzo Leso, Bernadette O’Brien, Christina Umstätter

**Affiliations:** 1 Agroscope, Tanikon 1, 8356 Ettenhausen, Switzerland; christina.umstaetter@agroscope.admin.ch; 2 Animal Nutrition and Rangeland Management in the Tropics and Subtropics, University of Hohenheim, 70599 Stuttgart, Germany; jessica.werner@uni-hohenheim.de; 3 Teagasc, Animal & Grassland Research and Innovation Centre, Moorepark, Fermoy, Co. Cork P61 C996, Ireland; emer.kennedy@teagasc.ie (E.K.); bernadette.obrien@teagasc.ie (B.O.); 4 Department of Agricultural, Food and Forestry Systems, University of Florence, 50145 Firenze, Italy; lorenzo.leso@unifi.it

**Keywords:** machine learning, binary classification, herbage allowance, feeding behaviour and activities, precision pasture management

## Abstract

Sensor technologies that measure grazing and ruminating behaviour as well as physical activities of individual cows are intended to be included in precision pasture management. One of the advantages of sensor data is they can be analysed to support farmers in many decision-making processes. This article thus considers the performance of a set of RumiWatchSystem recorded variables in the prediction of insufficient herbage allowance for spring calving dairy cows. Several commonly used models in machine learning (ML) were applied to the binary classification problem, i.e., sufficient or insufficient herbage allowance, and the predictive performance was compared based on the classification evaluation metrics. Most of the ML models and generalised linear model (GLM) performed similarly in leave-out-one-animal (LOOA) approach to validation studies. However, cross validation (CV) studies, where a portion of features in the test and training data resulted from the same cows, revealed that support vector machine (SVM), random forest (RF) and extreme gradient boosting (XGBoost) performed relatively better than other candidate models. In general, these ML models attained 88% AUC (area under receiver operating characteristic curve) and around 80% sensitivity, specificity, accuracy, precision and F-score. This study further identified that number of rumination chews per day and grazing bites per minute were the most important predictors and examined the marginal effects of the variables on model prediction towards a decision support system.

## 1. Introduction

One of the key roles of precision pasture management is to ensure that the herbage allowance is well maintained and utilised for the individual cows through the applications of smart farming technologies. In order for economical and efficient usage of the technologies, it is extremely important that the procedure analyses the recorded data to assist farmers in diverse decision-making processes. The RumiWatchSystem, consisting of a noseband pressure sensor [1] and a pedometer [2], is such a sensor-based technology in which the physical activities as well as grazing and ruminating behaviour of individual cows can be recorded. The reliability and validity of sensor data and their applications in precision farming were studied in a wide range of literature. For example, Greenwood, et al. [3] proposed simple initial algorithms for predicting pasture intake by individual cattle using sensor data. Other studies (e.g., [4,5]) addressed the scope of developing the support systems that could assist farmers with proper feed allowances, physical activities and behavioural changes, estimation of herbage dry matter and locomotion behaviour of the cattle.

In a similar context, the present study considers the problem of identifying the cows with insufficient herbage allowance based on a set of RumiWatchSystem recorded variables. Since direct measurement of herbage intake of cows on pasture is difficult, time consuming and expensive, this study explored the scope of using the variables as predictors of a decision class in binary classification, i.e., sufficient or insufficient herbage allowance. The data were collected from a study where a group of spring calving dairy cows had access to 100% of their intake capacity as herbage allowance, whereas another group had 60% of their intake capacity [6]. Each cow was equipped with an automated noseband pressure sensor and a pedometer, which continuously recorded the feeding and activity related variables. For the present study, the recorded variables were summarised (total or mean) to extract the features in 24-h windows. The rationale of this study lies in the fact that the complexities of herbage intake measurements can be reduced substantially if a classification model is found that efficiently predicts the insufficient allowance using the extracted features, towards a decision support system for optimal pasture management.

The subsequent sections of this article are organised as follows. The datasets used in this study, exploratory analysis for variable selection, commonly used machine-learning (ML) models in R [7] and the performance metrics used for evaluating and comparing the models are discussed in Section 2. Section 3 demonstrates the results of validation studies for the commonly used ML models and generalised linear model (GLM). This section further identifies the important variables, observed thresholds and the marginal effects of the variables on the model prediction. Section 4 discusses the study findings followed by a summary of this article in Section 5.

## 2. Materials and Methods

### 2.1. Data Collection

Data were collected for this study from a larger overall experiment at Teagasc, Moorepark Dairy Research Farm, Animal & Grassland Research and Innovation Centre, Fermoy, Co. Cork, Ireland. The experiment was conducted in spring time 2016 using 105 calving cows to examine the effects of restricted herbage allowance on milk production, immunology and indicators of reproductive health of grazing dairy cows. Ethical approval was received from Teagasc Animal Ethics Committee (TAEC; TAEC100/2015) and the procedure authorisation was granted by the Irish Health Products Regulatory Authority (HPRA).

For the present study, 40 focal cows were selected for recording the feeding behaviour and activities using the RumiWatchSystem. Out of these, 10 cows were randomly selected to have 100% of their intake capacity. The remaining 30 cows had restricted herbage allowance, i.e., 60% of their intake capacity. The 60% group was further divided into six blocks with respect to the period of restriction (two-week or six-week) and stages of lactation at the commencement of restriction: start (S: restriction started at the beginning of experiment), mid (M: two weeks after the S restriction commenced) or late (L: four weeks after the S restriction commenced). The behaviour of cows in the 100% group was monitored over a 10-week period. The three blocks S2, M2 and L2, which received two-week restriction of herbage allowance, had their behaviour recorded during the full two-week period, whereas the behaviour of blocks M6 and L6, which received six-week restriction, were recorded during the last two weeks of the restriction period. The S6 block was monitored during the entire six-week restriction period in order to mitigate the imbalance frequency of rows for the 100% and 60% groups in the combined data.

The RumiWatchSystem recorded pressure and accelerometer data in a 10 Hz resolution. The raw data were then converted into one-hour summaries by generic algorithms included in the RumiWatch Converter V.7.3.36, which were later summarised in individual daily records (features) per animal. There was some data loss and changing cows due to injuries and breakdown of sensors. As a result, there were 63 individual daily records per cow in the 100% group over a 10-week period included and 12 or 13 daily records per individual cow in the 60% group (except S6 block) depending on the application time of the sensor, as only complete daily records during the two-week period were considered. Only two cows had less than 12 daily records, due to technical issues with the sensor device. In case of S6 block, there were 38 individual daily records for four cows and 36 daily records for one cow during the six-week period included. The missing and incomplete rows were removed for the safety and strictness in comparing the prediction performance of the competing models.

Thus, the combined dataset included 1096 rows and 21 columns with 629 rows for the cows with 100% herbage allowance and 467 rows for the cows with 60% allowance. Each column included the extracted features (daily mean or total) of individual cows based on the recorded feeding behaviour or activity related variable. Out of the 21 features (variables), those listed in Table 1 were, on average, significantly different in the 100% and 60% allowance groups, hence considered as model predictors in this study. The study design is further discussed in [6].

The combined dataset were divided into six subsets based on the blocks of cows in the 60% allowance group. Throughout this paper, S2, S6, M2, M6, L2 and L6 denote the blocks of cows with restricted allowance as well as the datasets, which contained the respective rows from the 60% and 100% herbage allowance groups. In addition, the 100% and 60% groups are called *sufficient allowance* and *insufficient allowance* in the prediction of decision classes. The S2, M2 and L2 datasets were merged to create W2, which comprised the recorded features for two-week duration. Similarly, S6, M6 and L6 datasets were merged to create W6. These additional subsets of combined data were used to compare the changes in prediction performance as the duration of 60% herbage allowance increased from two to six weeks, regardless the lactation stages of the cows. Thus, the number of rows which corresponded to the cows with unrestricted and restricted allowance in the subsets S2, S6, M2, M6, L2 and L6 were (130, 65), (130, 65), (119, 60), (130, 56), (130, 52), and (120, 38), respectively.

In the present study, a number of predictive models were first applied to the combined data and the performance was compared based on leave-out-one-animal (LOOA [8]) approach to validation and cross validation (CV) studies. The models were further compared using the subsets of combined data based on CV studies.

### 2.2. Variable Selection

**Figure 1:**
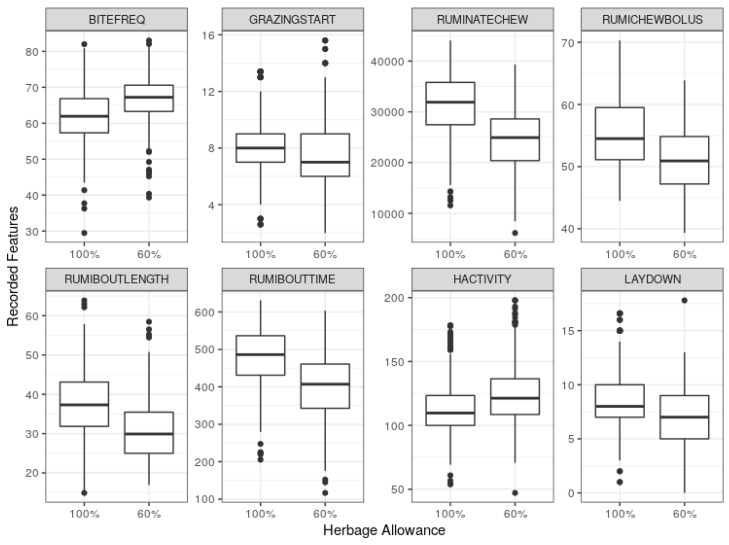
Side-by-side box plots of selected variables using the combined data for sufficient (100%) and insufficient (60%) herbage allowance groups.

A set of predictor variables was selected based on the exploratory analysis, i.e., box plots (Figure 1), *t*-tests (Table A1 and Table A2) and analysis of variance (Table A3). The selected variables were broadly classified as grazing behaviour, rumination behaviour and activity. The definitions, measurement units and notations used to denote the variables are presented in Table 1. For each variable, the measurement unit indicates the extracted feature (using 24-h window) considered in this study. Throughout this paper, the variable names will refer to the corresponding features extracted from the sensor data.

On average, the RumiWatchSystem-recorded measures of these variables in the sufficient allowance group was significantly different from at least one of the blocks of insufficient allowance group. For example, using the combined data, the side-by-side box plots in Figure 1 show that most of the selected variables centred higher in the 100% group than 60% group, except bite frequency per minute (BITEFREQ) and head activity index (HACTIVITY), which centred higher in the 60% group. In this study, GLM and ML models used these variables as predictors of the herbage allowance classes.

### 2.3. Classification Models

The commonly used ML models and GLM with binomial family were considered for the binary classification problem. For convenience, the dependent variable *herbage allowance* is denoted by *y* where y=1 and 0 refer to the insufficient and sufficient herbage allowance class, respectively. Given a set of predictor variables *X* for *n* observations, the GLM with logit link (Equation (1)) predicts insufficient herbage allowance if the estimated logit, $log(\pi_{i}/\left(1-\pi_{i}\right))>0$ or sufficient allowance if $log(\pi_{i}/\left(1-\pi_{i}\right))<0$.

(1) $$log(\frac{\pi_{i}}{1-\pi_{i}})=X\beta .$$

Here, $\pi_{i}=p\left(y=1\right)$ denotes the probability of insufficient allowance and $1-\pi_{i}=p\left(y=0\right)$ denotes the probability of sufficient allowance for the *i*th observation (i=1,2,…,n). The GLM was implemented using the glm function of the stats package in R [7]. Table 2 presents the list of ML methods considered in this study, and the packages that implement the methods in R. In each case, the underlying classification model used the variables of Table 1 as predictors. For more details and familiarising with hyper-parameters of specific ML, see the R package caret [10].

In this study, first the performance of the ML models and GLM was compared using combined data. At this stage, the predictive performance of the models was evaluated based on LOOA approach and CV studies. Then, GLM and selected ML models, which achieved desirable performance, were further compared based on CV studies using S2, M2, L2, S6, M6, L6, W2 and W6 datasets. Thus, the effect of restriction period on predictive performance was examined for separate blocks and regardless the lactation stages of the calving cows. Finally, the important variables and partial dependencies of model prediction were examined for random forest (RF).

### 2.4. Evaluation Metrics

The prediction performance of the candidate models was compared based on a number of classification evaluation metrics. The metrics were estimated in validation studies using the confusion matrix (Table 3) of actual and predicted classes for the test cases. Table 4 shows the estimation formulae for the list of metrics considered in this study.

For binary classification, one way to evaluate the performance of a predictive model is the estimation of accuracy, i.e., the rate of correctly predicting the class of a test case. Accuracy is a commonly used evaluation metric since it takes into account both true negative and true positive rates. Here, negative means sufficient allowance and positive means insufficient allowance. However, in the case of imbalance training data, accuracy is often overestimated. The area under receiver operating characteristic curve (AUC [16]) also considers true negative and true positive rates and is often used along with other evaluation metrics. In the context of present study, AUC denotes the probability that a randomly chosen cow with insufficient allowance is ranked higher than a cow with sufficient allowance. Both accuracy and AUC range in value from 0 to 1, a higher value indicating greater ability to discriminate one class from the other. According to Steensels, et al. [17], a diagnostic test is usually classified as excellent (AUC = 0.9–1), good (AUC = 0.8–0.9), fair (AUC = 0.7–0.8), poor (AUC = 0.6–0.7) or fail (AUC = 0.5–0.6).

Since the subsets of the combined data were unbalanced, accuracy and AUC were not sufficient in this study to validate the performance of the competing models.

Moreover, in the case of an animal monitoring model, it is often more important to identify cows with insufficient feed allowance than sufficient allowance. Thus, additional metrics, namely specificity, sensitivity, positive predictive value (PPV) and F-score, were considered in this study. Here, specificity (rate of predicting sufficient allowance given the cow had sufficient allowance) assesses the prediction performance for the test cows in the 100% herbage allowance group. Conversely, sensitivity, PPV and F-score focus on the correct prediction rate for cows with insufficient herbage allowance. Sensitivity of a model estimates the rate at which insufficient allowance was predicted when a randomly selected cow actually had 60% allowance. The PPV metric further estimates the proportion of predicted insufficient allowance that were actually insufficient. The F-score considers both sensitivity and PPV since it is the harmonic mean of these two metrics. Thus, a high F-score implies that the model is highly efficient in predicting insufficient herbage allowance.

In this study, the performance of the candidate models was compared based on the estimates of these metrics using validation studies. For the combined data, the estimates were first obtained based on LOOA approach, where data from one animal create the test set while the remaining animals create the training set. Since the candidate models are trained with no overlapping features that come from the same animal in the test set, the LOOA approach gives the estimated metrics that are more reliable in the prediction of new (unseen) animal. However, in the present context, since the previous data of cows on pasture can be included in the training set, the evaluation metrics were further estimated based on CV studies. This approach identified the models, which may perform relatively better when a support system continuously updates the training data with the previous records of cows on pasture. Given a dataset, the CV study was conducted as follows.

i. Randomly split the observations into a training and a test set such that each observation has 70% chance to be included in the training set and 30% chance to be included in the test set.
ii. Train the ML models (fit the GLM) in the training set and apply them for predicting the herbage allowance classes in the test set.
iii. Create a confusion matrix for each model and estimate the evaluation metrics of Table 4.
iv. Repeat Steps i–iii a large number (1000) of times and summarise the results by the mean and standard error of the estimates for each model.

## 3. Results

### 3.1. Predictive Performance

Table 5 and Table 6 summarise the results for combined data using LOOA approach to validation and CV studies. In LOOA approach, since the estimates were obtained by using a single confusion matrix for all calving cows under study, the standard errors of the estimates were not applicable.

It can be observed that in both LOOA and CV studies the ML models predicted the sufficient and insufficient allowance classes relatively more accurately than GLM. Table 5 reveals that, on average, the prediction accuracy of insufficient allowance using linear discriminant analysis (LDA) (78% sensitivity) and that of sufficient allowance using naïve Bayes (NB) (74% specificity) were higher than all other models. Additionally, the NB model attained relatively higher prediction accuracy (73%), PPV (68%), F-score (70%) and AUC (81%), which indicate that the model can be more reliable in predicting the herbage allowance classes of new calving cows based on the current data. The neural network (NNET) and GLM also attained the F-scores equal 70%. The more advanced ML models such as RF and XGBoost attained similar accuracy when predicting the insufficient allowance but relatively lower accuracy when predicting the sufficient allowance in LOOA approach. The sensitivity, specificity, accuracy, PPV, F-score and AUC estimates of random forest (RF) model were 75%, 63%, 68%, 60%, 67% and 76%, respectively. Comparing the results in Table 6, it is further observed that there was an increase in the estimated metrics of each model when a portion of features in the training and test set were observed from the same cows. This indicates that the models were over trained in CV approach, i.e., the estimates may be reliable in case the future prediction of herbage allowance is based on previous records of the cows included in the training set. Using CV approach, the support vector machine (SVM), extreme gradient boosting (XGBoost) and RF models achieved relatively higher accuracy (≈80%) and AUC (88%) than GLM and other ML models. The observed accuracy and AUC for GLM were 76% and 85%. Comparing the sensitivity, specificity, PPV and F-score, the SVM, XGBoost and RF models, on average, scored higher values (≈80%), whereas the estimates for other ML models lied mostly in the range 70%–78%. GLM attained these estimates around 76%. The standard errors of the estimates were small in CV studies, which indicate that the estimates were precise. Based on the results in Table 5 and Table 6, GLM, RF, XGBoost, SVM, LDA, NNET, and NB models were selected for CV studies using the subsets of combined data.

### 3.2. Effects of Restriction Period

Table 7 and Table 8 summarise the CV results for GLM and RF using the subsets of combined data. Similar tables are created for SVM, XGBoost, LDA, NNET and NB in the Appendix A (Table A4, Table A5, Table A6, Table A7 and Table A8).

Using these tables, the relative predictive performance can be compared for two-week and six-week restriction periods. Thus, the effect of restriction period on the underlying models can be examined by comparing the pairs of rows (S2, S6), (M2, M6), (L2, L6) and (W2, W6). For example, the S2 and S6 rows indicate the changes in the estimated metrics due to a relatively longer period of insufficient herbage allowance for the cows in an early stage of lactation. Similarly, M2 and M6 rows indicate the effects of restriction period for the cows in a mid stage of lactation, and L2 and L6 rows indicate the effects for the cows in a late stage of lactation at the commencement of restricted allowance. The additional rows, W2 and W6 compare the overall effect of a longer restriction period on the predictive performance, regardless the lactation stages.

In Table 7, it can be observed that GLM achieved more than 80% specificity, accuracy and AUC in most cases. The high specificity estimates indicate that if a randomly selected cow had sufficient herbage allowance, GLM would predict sufficient allowance with a rate higher than 80%. However, the estimated sensitivity, PPV and F-score of GLM were relatively low. Unlike S and L, the estimates for block M decreased with the increase of restriction period to six weeks. This indicates that the effect of restriction period on the performance metrics of GLM was not consistent with all lactation stages. The W2 and W6 rows of Table 7, however, reveal that the overall effect of restriction period on GLM based prediction was negative, since the predictive performance decreased for six-week restriction period.

The results for RF (Table 8), SVM and XGBoost (Table A4 and Table A5) were different from GLM in that, the performance metrics increased in most cases as the restriction period increased from two weeks to six weeks. For the XGBoost and RF models, the effect of restriction period was similar in the S, M and L blocks. While the estimates for SVM were, in general, higher than GLM, the XGBoost and RF models were more consistent and performed relatively better than SVM in most cases. The estimated sensitivity of the RF model was at least 87% for each lactation stage. Likewise, the PPV and F-score estimates were close to 80% or higher in most cases. However, these estimates decreased and lied in the range 60%–80% for W2 and W6 data. Nonetheless, the correct prediction rate of insufficient allowance by the RF model was higher than all other models. Table 8 further reveals that the estimated metrics based on the S6, M6 and L6 data were no less than the estimates based on the S2, M2 and L2 data. Thus, the CV studies demonstrated that the effect of restriction period on the performance of RF model was consistent with the lactation stages. The XGBoost model performed similarly as the RF model in most cases (Table A5). As with CV approach for combined data, the LDA, NNET and NB models attained relatively lower values of the estimates (Table A6, Table A7 and Table A8) in the separate analyses, especially in case of predicting insufficient allowance class. Based on the validation results in this study, it can be concluded that, apart from high (>80%) specificity, accuracy and AUC in all cases, the RF and XGBoost models maintained a nice balance in correct prediction rate of sufficient and insufficient herbage allowance using CV approach, hence preferred to other candidate models in the present context.

### 3.3. Partial Dependencies on Important Variables

**Figure 2:**
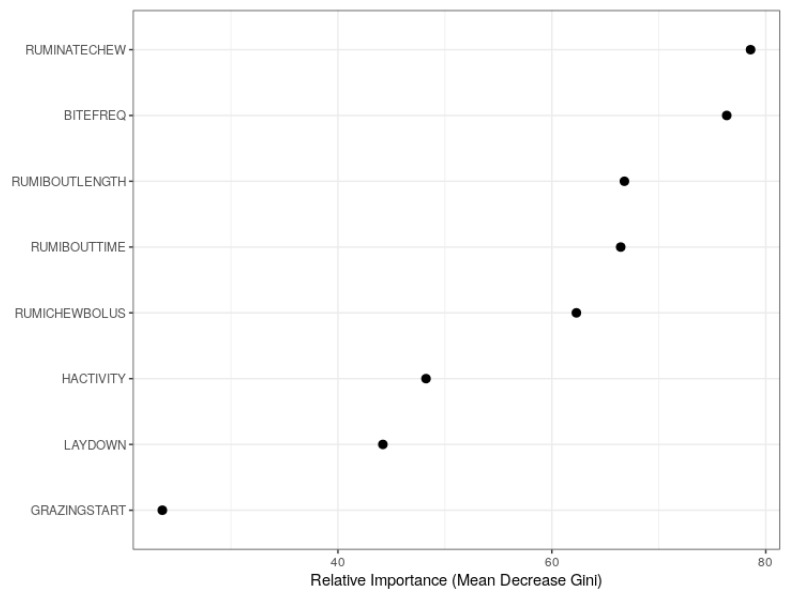
Variable importance plots for random forest based on mean decrease of Gini coefficients.

**Figure 3:**
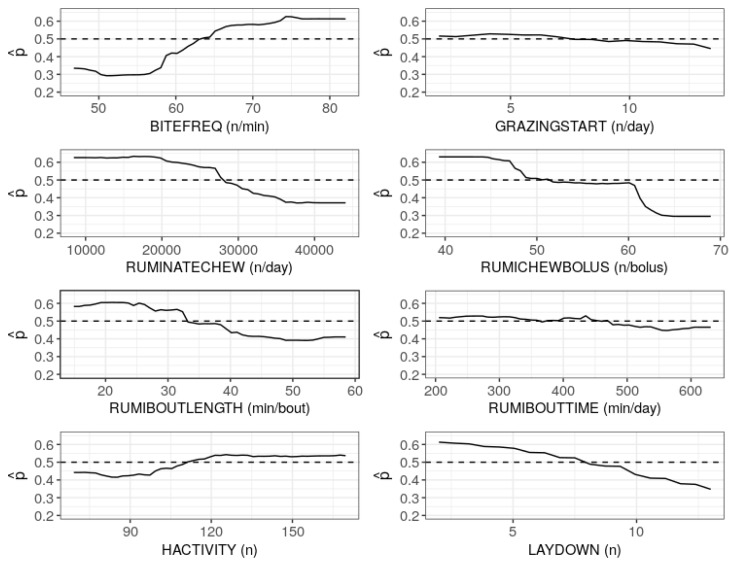
Partial dependence plots for the marginal effects of the predictors on the random forest model: Estimated probability of insufficient allowance versus the observed values of each predictor in the combined data.

Figure 2 shows the relative importance of the predictor variables using RF. The predictors are plotted in order of rank against the mean decrease of Gini coefficients. It is observed that the number of rumination chews per day (RUMINATECHEW), BITEFREQ, mean duration of rumination bout (RUMIBOUTLENGTH), rumination time within all rumination bouts (RUMIBOUTTIME) and mean number of rumination chews per bolus (RUMICHEWBOLUS) were relatively more important for the prediction of herbage allowance. The importance plots in Figure 2 were not sufficient since it was not clear which predictors had positive and negative effects on the model prediction. The RF model has an advantage since it allows graphical examination of partial dependencies of the model on each predictor. Figure 3 shows the partial dependence plots (PDP [18]) using the probability of insufficient herbage allowance (decision class). Here, the estimated probabilities ($\hat{p}s$) of the decision class were plotted against the observed values of the predictor variables.

Thus, the PDPs indicate how the variables marginally affected the prediction based on RF model. Assuming all other variables fixed at the centre, the values of a given predictor that correspond to the probability higher than 0.5 indicate a positive effect and the values that correspond to the probability lower than 0.5 indicate a negative effect on the prediction. The values on the *x*-axis which correspond to $\hat{p}\approx 0.5$ imply that the sufficient and insufficient classes are not distinguishable (i.e., both the classes are equally likely). This implies that the predictor may not have noticeable marginal effect on the model if the PDP lies near 0.5 over the range values on the *x*-axis. Figure 3 reveals that the marginal effects of number of grazing bouts started per day (GRAZINGSTART) and time of rumination within all rumination bouts (RUMIBOUTIME) were not significant, whereas the remaining variables had noticeable marginal effects on the prediction based on RF model.

Intuitively, the cut-off point on the *x*-axis which corresponds to the unique intersection point of PDP and the horizontal dashed line ($\hat{p}\approx 0.5$), indicates that the model declares insufficient allowance and sufficient allowance for the values of the predictor that lie in the opposite direction of the cut-off point. In particular, Figure 3 suggests that for a given predictor, insufficient allowance was more likely than sufficient allowance when $\hat{p}>0.5$, and it was less likely than sufficient allowance when $\hat{p}<0.5$. In the present study, thus the RF model tended to declare insufficient herbage allowance for most of the predictors being lower than the cut-off points, except BITEFREQ and HACTIVITY. While BITEFREQ showed increasing positive effect for values higher than the cut-off point, the positive effect of HACTIVITY gradually decreased near 200. Moreover, RUMICHEWBOLUS had positive effects in the range <50/bolus, negative effects in the range >60/bolus and no noticeable effects in the range 50–60/bolus.

### 3.4. Thresholding the Predictors

**Figure 4:**
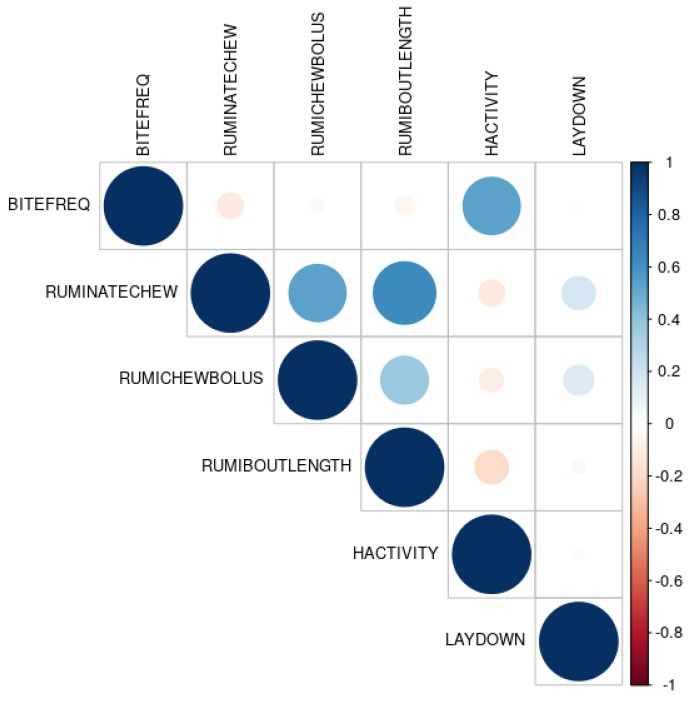
Pairwise correlations among the important predictors in the random forest model. Intensity of blue and red colour represents the strength of positive and negative correlation, respectively.

The cut-off points on the *x*-axis of PDPs suggest the observed thresholds for the predictors that marginally discriminate the prediction of insufficient herbage allowance from the sufficient allowance. However, the PDPs assume that the predictors are not correlated. Violation of this assumption may result in biased marginal effects and cut-off points, since this often lead the data points to occur in the areas of the distribution where the actual probability is very low. This complicates the interpretation of partial dependencies and may result in misleading thresholds. Based on the variable importance plots and PDPs, the pairwise correlations among the important predictors are plotted in Figure 4.

**Figure 5:**
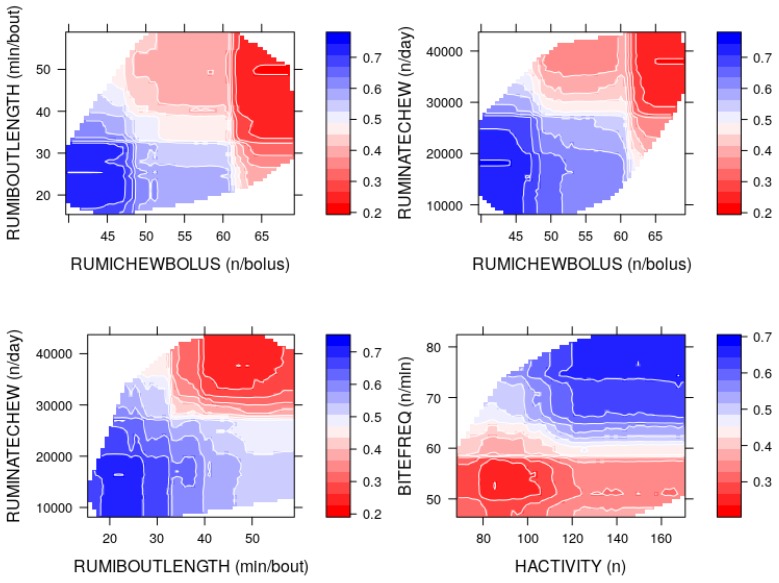
Contour plots for the partial dependencies of random forest on correlated predictors: Colour intensity represents the estimated probability of insufficient allowance versus the recorded predictors in the combined data.

It can be seen that the pairs (RUMINATECHEW, RUMIBOUTLENGTH) and (BITEFREQ, HACTIVITY) were moderate to highly correlated, and the pairs (RUMINATECHEW, RUMICHEWBOLUS) and (RUMICHEWBOLUS, RUMIBOUTLENGTH) were weak to moderately correlated. One approach to simultaneously studying the marginal effects of two correlated variables is to plot the estimated probabilities in a contour plot as shown in Figure 5.

Here, colour represents the intensity of effects on the model due to simultaneously changing the predictor variables on the *x*- and *y*-axis. The prediction of the RF model was *insufficient herbage allowance* in the dark blue area and *sufficient herbage allowance* in the dark red area. In the range from light red/blue to white area, the predictions would be similar to random guesses, hence not reliable.

Based on the contour plots and PDPs, the estimated ranges of predictor values which correspond to p(y=0)>0.5 and p(y=1)>0.5 are presented in Table 9. The observed thresholds are approximate (using set.seed(8356) in R) since the RF algorithm randomly selects a number of rows and columns for training sets, which may result in slightly different values each time the model is run.

Nonetheless, Table 9 exhibits heuristically the predictor values at which the RF model tended to predict the sufficient and insufficient herbage allowance for the spring calving dairy cows under study. For example, given all other predictors fixed at the centre, the RF model would predict insufficient allowance if the RumiWatchSystem recorded BITEFREQ in the range 64–82/min and HACTIVITY index in the range 112–170. Similarly, all other thresholds can be interpreted. It is important to note that the ranges of RUMIBOUTTIME which corresponded to $\hat{p}\left(y=0\right)>0.5$ and $\hat{p}\left(y=1\right)>0.5$ were not distinct. Thus, RUMIBOUTTIME exhibited no significant marginal effect in the predictive performance of the RF model.

## 4. Discussion

The results of LOOA and CV approaches for combined data identified a set of ML models, which achieved relatively higher accuracy than GLM. The observed differences in the estimates using the two approaches indicate that while most ML models and GLM may be equally reliable in predicting the insufficient allowance of new calving cows, the SVM, XGBoost and RF models may perform relatively better, when the previous records of cows on pasture can be included in the training set. Since the aim of this study is to assist developing a support system, which continuously updates the data of all cows, in the present context, it is more practical that a portion of overlapping features in the training and test set may come from the same cows. Thus, the present study highlights validation of model performance based on CV studies.

The results of CV studies demonstrate that RF and XGBoost out performed GLM and all other ML models in predicting both sufficient and insufficient allowance classes. The SVM model also showed desirable performance in most cases. NNET is one of the most popular ML methods, which performs well for large and complex datasets. However, the present study involved a relatively small dataset and applied a simple (single layer) NNET due to an insufficient training set for a more sophisticated NNET. The single layer NNET performed similar to GLM, LDA, and NB models but did not perform as good as RF or XGBoost in CV studies.

The separate CV studies using the subsets of combined data indicate that the predictive performance was affected by the duration of restricted allowance among the 60% herbage allowance groups. Intuitively, if the restricted herbage allowance affects the feeding behaviour and activities, it is reasonable to assume that, in general, cows with a longer restriction period would exhibit a greater difference from the unrestricted group than those with a shorter restriction period. Thus, a good predictive model would distinguish the herbage allowance classes more efficiently when applied to the test cases from S6, M6, L6 and W6 data compared to S2, M2, L2 and W2 data. In this study, it was demonstrated that the estimated performance metrics for the RF and XGBoost models were consistently higher in cases of longer restriction periods.

Additionally, the ML methods have advantage over GLM since the underlying models consider nonlinear relationships and do not rely on strict assumptions. Rather the algorithms learn from the training datasets, develop a classification rule based on the learning and validate the rule to the unseen cases before generalising the model for applications to the new cases. For example, the decision tree (DT) model learns how to best split the dataset into smaller and smaller subsets for predicting the target classes. The splitting process continues until no further knowledge gain can be made or a pre-set rule is met (e.g., reaches the maximum depth of the tree). The learning process of DT is further improved in more advanced and efficient algorithms such as the RF and XGBoost algorithms, which build multiple DTs from randomly selected subsets of the training set and merge the knowledge together to generate a final model. Thus, RF and XGBoost usually achieve greater accuracy and stable prediction as shown in CV studies. However, in case of LOOA approach, these models performed similar to other ML models and GLM in predicting insufficient allowance class but attained relatively lower specificity. Since our specific aim in this study is to assist creating a decision support system, which may include the previous records in training the models, and identify cows with insufficient allowance for farmers, the CV approach further demonstrated that the additional data improved the prediction performance of RF, XGBoost and SVM, relatively better than all other candidate models.

Using CV studies the estimated AUC of the RF, XGBoost and SVM models was above 90% in most cases, which indicate that these models, in general, achieved excellent classification performance. The results from the combined data further show that the estimates of all other metrics were close to 80% or higher. Using the subsets of combined data, while the estimated specificity was more than 80% in all cases, the sensitivity estimates were relatively low using W2 and W6 data. Moreover, the PPV and F-score estimates for the RF and XGBoost models were higher than SVM in all subsets. One possible reason for the alterations in the results for W2–W6 data and separate blocks can be the effect of lactation stages, i.e., the variation of predictors among the lactation stages in the combined datasets. In general, the RF, XGBoost and SVM model showed relatively better performance in the separate analyses using the pairs (S2, S6), (M2, M6) and (L2, L6) compareed to the merged datasets W2 and W6.

In practice, since the duration of insufficient allowance is usually unknown, the relative importance and marginal effects of the predictors were studied using the combined data. The importance plots indicated that the number of rumination chews per day, grazing bites per minute, mean duration of a rumination bout, time of rumination within all rumination bouts and mean number of rumination chews per bolus were relatively more important predictors. The partial dependence plots further revealed that grazing bites per minute and head activity index had positive marginal effects while the number of rumination chews per day, mean number of rumination chews per bolus, mean duration of a rumination bout and standing or lying frequency index had negative marginal effects on the RF model. The effects of number of grazing bout starts and time of rumination within all rumination bouts were not significant.

As the correlation among the important predictors was taken into account, the contour plots further revealed the observed ranges for the correlated predictors, at which the RF model was more likely to declare sufficient and insufficient herbage allowance class. It was observed that the RF model would predict *insufficient allowance* when the RumiWatchSystem recorded higher values for BITEFREQ >64/min) and HACTIVITY (>111), and lower values for RUMINATECHEW (>27,685/day), RUMICHEWBOLUS (>50/bolus), RUMIBOUTLENGTH (>32 min/bout), LAYDOWN (>7) and GRAZINGSTART (>7/day).

As one of the key roles of precision pasture management is to ensure that herbage allowance is well maintained and utilised for the individual cows, our findings have important implications in the quest to develop precise and reliable decision support systems for pasture management in order to assist farmers. With growing consumer demands for animal welfare [19] and the worldwide human population increase [20], there is pressure on farmers to optimally utilise the world’s grasslands. Since grassland is heterogenic, herbage growth is almost unpredictable, and individual feed intake differs between cows, pasture management is difficult and laborious. However, at the onset of pasture management, farm staff know that the cows on pasture have enough herbage to cover their requirement. It can therefore be of great help for farmers to detect the point of change from sufficient to insufficient pasture allocation for the individual cows. As the support system is aimed to regularly update the behavioural data, the current records can be added to improve the allocation prediction. Thus, all the previously recorded features of the cows feed into the model for predicting their decision classes. In this context, the cross-validation results in this study indicate that a decision support system using the RF and XGBoost models could correctly predict the sufficient or insufficient allowance of the cows at a rate around 80% or higher including the different subsets.

In a real world system, the observed thresholds may be useful for prediction (i.e., the current data can be used as training set) under the assumption that the cows on pasture are similar, the recorded features lie within the observed ranges, and the extraneous factors such as temperature, climate condition, pasture condition, grass quality, etc. are also similar to the ones in this study. However, it is important to note that the thresholds are approximate since the underlying algorithms were trained by the randomly selected subsets of the data used in this study. In general, care needs to be taken while applying the thresholds for future predictions. Since the present study identified more than one models that attained relatively higher accuracy in different conditions, it is further recommended to apply GLM, NB, LDA and NNET along with SVM, XGBoost and RF, and determine the decision class for new calving cows based on majority voting. In the case of different environment, pasture conditions or different cow breeds, the models should be trained with new datasets and checked for validity of the observed thresholds. Nonetheless, the results obtained in this study provide a strong foundation towards ML based predictions of insufficient herbage allowance through decision support systems in precision pasture management. Especially, the methods RF and XGBoost have shown their strength in the context of present study, across the different subsets of data and are, therefore, particularly well-suited for a decision support system.

## 5. Conclusions

The results of this work demonstrate that a set of RumiWatchSystem recorded feeding behaviour and activity related variables could be used to predict insufficient herbage allowance of spring calving dairy cows. Along with naïve Bayes, linear discriminant analysis and neural network, the prediction based on random forest and extreme gradient boosting could be similar or more reliable than GLM and other commonly used models in machine learning. The predictive performance of these models was affected by the period of restricted herbage allowance. In general, insufficient allowance was correctly predicted at a higher rate in case of six-week restriction periods than two-week restriction periods. Based on the graphical presentation of marginal effects, the RF model further suggested the ranges of predictor values, at which the model was apt to declare sufficient or insufficient herbage allowance to be the decision class. The next step would examine the validity of these thresholds as well as the performance of the proposed models for similar studies in other pasture management systems towards developing a decision support system.

## Figures and Tables

**Table 1:** List of feeding behaviour and activity related variables used in the classification models.

Notation	Grazing Behaviour
BITEFREQ	Bite frequency or grazing bites per min (n/min)
GRAZINGSTART	Number of grazing bouts started per day (grazing bout = minimum duration of 7 min and intra-bout interval is smaller than 7 min [9]) (n/day)
	**Rumination Behaviour**
RUMINATECHEW	Number of rumination chews per day (n/day)
RUMICHEWBOLUS	Mean number of rumination chews per bolus (n/bolus)
RUMIBOUTLENGTH	Mean duration of a rumination bout (rumination bout = minimum duration of 3 min and intra-bout interval is smaller than 1 min [9]) (min/bout)
RUMIBOUTTIME	Time of rumination within all rumination bouts (min/day)
	**Activity**
HACTIVITY	Head movement activity index (n) based on accelerometer data; the averaged variance of 3-dimensional acceleration captured on the head in 10-s segments
LAYDOWN	Number of event (n) at which the pedometer angle changes its position from a vertical angle towards a horizontal angle for a duration of at least 50 s when the cow is lying down or standing up [2]

**Table 2:** List of machine learning methods with R packages.

Machine Learning	R Package	Function(s)
k-Nearest Neighbour (kNN)	class [11]	knn
Linear Discriminant Analysis (LDA)	MASS [11]	lda
Neural Network (NNET)	nnet [11]	nnet
Naïve Bayes (NB)	e1071 [12]	naiveBayes
Support Vector Machine(SVM)	e1071 [12]	svm
Decision Tree (DT)	rpart [13]	rpart
Random Forest (RF)	randomForest [14]	randomForest
Extreme Gradient Boosting (XGBoost)	xgboost [15]	xgb.DMatrix, xgb.train

**Table 3:** Confusion matrix for estimating the classification evaluation metrics based on the number of actual and predicted classes among the test cases.

Predicted Herbage Allowance	Allocated Herbage Allowance	
	Insufficient	Sufficient
Insufficient	True Positive (TP)	False Positive (FP)
Sufficient	False Negative (FN)	True Negative (TN)

**Table 4:** Estimators of sensitivity, specificity, accuracy, positive predictive value (PPV), F-score and the area under receiver operating characteristic curve (AUC) in terms of the number of true positive (TP), false positive (FP), true negative (TN) and false negative (FN) classes among the test cases.

Evaluation Metric	Estimator
Sensitivity	$\frac{TP}{TP+FN}$
Specificity	$\frac{TN}{TN+FP}$
Accuracy	$\frac{TN+TP}{TP+FP+TN+FN}$
Positive predictive value (PPV)	$\frac{TP}{TP+FP}$
F-score	$\frac{2\times Sensitivity\times PPV}{Sensitivity+PPV}$
AUC	Area under ROC curve

**Table 5:** Predictive performance of machine learning and generalised linear models based on the estimated sensitivity, specificity, accuracy, positive predictive value (PPV), F-score and the area under receiver operating characteristic curve (AUC) using leave-out-one-animal approach to validation studies for combined data.

Classifier	Sensitivity	Specificity	Accuracy	PPV	F-Score	AUC
kNN	0.70	0.71	0.71	0.64	0.67	0.78
NB	0.72	**0.74**	**0.73**	**0.68**	**0.70**	**0.81**
NNET	0.77	0.67	0.71	0.63	**0.70**	0.80
LDA	**0.78**	0.65	0.70	0.62	0.69	0.79
DT	0.74	0.67	0.70	0.63	0.68	0.78
SVM	0.74	0.61	0.67	0.59	0.66	0.74
XGBoost	0.73	0.59	0.65	0.57	0.64	0.72
RF	0.75	0.63	0.68	0.60	0.67	0.76
GLM	0.74	0.64	0.69	0.63	**0.70**	0.76

The estimates in bold correspond to the best models.

**Table 6:** Predictive performance of machine learning and generalised linear models based on the estimated sensitivity, specificity, accuracy, positive predictive value (PPV), F-score and the area under receiver operating characteristic curve (AUC) using cross validation studies for combined data.

Classifier	Sensitivity	Specificity	Accuracy	PPV	F-Score	AUC
kNN	0.66 (0.004)	0.67 (0.003)	0.67 (0.002)	0.65 (0.003)	0.65 (0.003)	0.73 (0.003)
NB	0.73 (0.004)	0.73 (0.003)	0.73 (0.002)	0.71 (0.004)	0.72 (0.003)	0.81 (0.003)
NNET	0.76 (0.004)	0.78 (0.004)	0.76 (0.002)	0.76 (0.004)	0.76 (0.003)	0.85 (0.003)
LDA	0.76 (0.003)	0.78 (0.004)	0.77 (0.002)	0.77 (0.004)	0.76 (0.002)	0.85 (0.002)
DT	0.75 (0.003)	0.76 (0.004)	0.75 (0.003)	0.74 (0.005)	0.74 (0.003)	0.83 (0.003)
SVM	**0.79** (0.004)	**0.80** (0.003)	**0.79** (0.002)	**0.79** (0.004)	**0.79** (0.002)	**0.88** (0.002)
XGBoost	**0.79** (0.003)	**0.81** (0.003)	**0.80** (0.002)	**0.80** (0.003)	**0.79** (0.002)	**0.88** (0.002)
RF	**0.80** (0.003)	**0.80** (0.003)	**0.80** (0.002)	**0.79** (0.004)	**0.79** (0.002)	**0.88** (0.002)
GLM	0.76 (0.004)	0.77 (0.003)	0.76 (0.002)	0.76 (0.004)	0.76 (0.003)	0.85 (0.003)

Standard errors are indicated in parentheses. The estimates in bold correspond to the best models.

**Table 7:** Predictive performance of generalised linear model based on the estimated sensitivity, specificity, accuracy, positive predictive value (PPV), F-score and the area under receiver operating characteristic curve (AUC) for two-week and six-week restriction periods among the cows in early (S), mid (M) and late (L) lactation stage using cross validation studies.

Subset	Sensitivity	Specificity	Accuracy	PPV	F-Score	AUC
S2	0.78 (0.01)	0.81 (0.006)	0.80 (0.006)	0.68 (0.013)	0.72 (0.01)	0.88 (0.007)
S6	0.84 (0.01)	0.88 (0.006)	0.86 (0.005)	0.81 (0.01)	0.82 (0.008)	0.94 (0.004)
M2	0.82 (0.012)	0.82 (0.007)	0.81 (0.006)	0.69 (0.014)	0.74 (0.01)	0.89 (0.007)
M6	0.78 (0.014)	0.80 (0.005)	0.79 (0.004)	0.62 (0.012)	0.68 (0.009)	0.87 (0.006)
L2	0.74 (0.015)	0.81 (0.007)	0.78 (0.006)	0.63 (0.016)	0.67 (0.012)	0.85 (0.008)
L6	0.81 (0.02)	0.84 (0.005)	0.83 (0.005)	0.67 (0.02)	0.72 (0.013)	0.90 (0.007)
W2	0.74 (0.008)	0.84 (0.004)	0.81 (0.003)	0.63 (0.008)	0.68 (0.006)	0.87 (0.004)
W6	0.71 (0.009)	0.85 (0.003)	0.81 (0.003)	0.61 (0.008)	0.65 (0.007)	0.86 (0.004)

Standard errors are indicated in parentheses.

**Table 8:** Predictive performance of random forest based on the estimated sensitivity, specificity, accuracy, positive predictive value (PPV), F-score and the area under receiver operating characteristic curve (AUC) for two-week and six-week restriction periods among the cows in early (S), mid (M) and late (L) lactation stage using cross validation studies.

Subset	Sensitivity	Specificity	Accuracy	PPV	F-Score	AUC
S2	0.90 (0.011)	0.87 (0.007)	0.88 (0.006)	0.78 (0.015)	0.84 (0.01)	0.96 (0.005)
S6	0.91 (0.008)	0.91 (0.005)	0.91 (0.004)	0.86 (0.01)	0.88 (0.006)	0.97 (0.002)
M2	0.89 (0.015)	0.87 (0.007)	0.88 (0.006)	0.79 (0.015)	0.83 (0.013)	0.95 (0.008)
M6	0.89 (0.009)	0.88 (0.004)	0.89 (0.004)	0.79 (0.011)	0.83 (0.007)	0.95 (0.002)
L2	0.87 (0.011)	0.89 (0.006)	0.88 (0.005)	0.79 (0.014)	0.82 (0.009)	0.95 (0.005)
L6	0.91 (0.015)	0.90 (0.005)	0.90 (0.005)	0.80 (0.02)	0.85 (0.013)	0.96 (0.005)
W2	0.78 (0.008)	0.84 (0.004)	0.82 (0.003)	0.62 (0.01)	0.68 (0.007)	0.88 (0.004)
W6	0.78 (0.006)	0.88 (0.003)	0.85 (0.002)	0.69 (0.009)	0.73 (0.006)	0.91 (0.002)

Standard errors are indicated in parentheses.

**Table 9:** Observed ranges of predictor values that correspond to the prediction of sufficient (y=0) and insufficient (y=1) herbage allowance by the random forest model.

Predictor Variables	$\hat{p}\left(y=0\right)>0.5$		$\hat{p}\left(y=1\right)>0.5$	
	Min	Max	Min	Max
BITEFREQ	46	63	64	82
GRAZINGSTART	8	13	2	7
RUMINATECHEW	28,397	44,062	8461	27,685
RUMICHEWBOLUS	61	69	39	50
RUMIBOUTLENGTH	33	58	15	32
RUMIBOUTTIME	367	631	205	469
HACTIVITY	69	110	111	170
LAYDOWN	8	13	2	7

## Abbreviations

The following abbreviations are used in this manuscript:

ML	Machine Learning
RF	Random Forest
XGBoost	Extreme Gradient Boosting
SVM	Support Vector Machine
kNN	k-Nearset Neighbour
NB	Näive Bayes
LDA	Linear Discriminant Analysis
NNET	Neural Network
DT	Decision Tree
LOOA	Leave-Out-One-Animal
CV	Cross Validation
TP	True Positive
FP	False Positive
FN	False Negative
TN	True Negative
AUC	Area Under the Curve
PPV	Positive Predictive Value
ROC	Receiver Operating Characteristic
PDP	Partial Dependence Plot
BITEFREQ	Bite Frequency
GRAZINGSTART	Grazing Start
RUMINATECHEW	Rumination Chews
RUMICHEWBOLUS	Rumination Chews per Bolus
RUMIBOUTLENGTH	Rumination Bout Length
RUMIBOUTTIME	Rumination Bout Time
HACTIVITY	Head Activity (index)
LAYDOWN	Laying Down

## Appendix A

**Table A1:** Independent samples *t*-tests for significant differences of means of each predictor in the 100% and 60% herbage allowance groups using S2, S6, M2, M6, L2 and L6 data.

Variable	S2	S6	M2	M6	L2	L6
BITEFREQ	6.35 ***	4.76 ***	3.48 *	5.04 ***	3.2 ***	3.07 ***
GRAZINGSTART	−0.97 **	−0.29	0.05	−0.68	−0.76 *	−0.21
RUMINATECHEW	−1689 *	−9169 ***	−7482 ***	−3969 ***	−8429 ***	−7218 ***
RUMICHEWBOLUS	−1.24	−8.12 ***	−2.87 ***	−3.06 ***	−3.71 ***	−4.95 ***
RUMIBOUTLENGTH	−3.47 ***	−10.90 ***	−7.18 ***	−6.04 ***	−7.12 ***	−9.0 ***
RUMIBOUTTIME	−12.76	−107.04 ***	−105.07 ***	−53.9 ***	−104.4 ***	−80.0 ***
HACTIVITY	13.81 ***	15.31 ***	6.71	17.9 ***	8.53 **	9.29 *
LAYDOWN	−2.09 ***	−1.48 ***	−2.45 ***	−1.32 **	−1.36 ***	−0.81

P-value: *** < 0.001; ** < 0.01; * < 0.05.

**Table A2:** Independent samples *t*-tests for significant differences of means of each predictor in the 100% and 60% herbage allowance groups using W2, W6 and combined data.

Variable	W2	W6	Combined
BITEFREQ	4.5 ***	4.22 ***	4.48 ***
GRAZINGSTART	−0.538 *	−0.343	−0.60 **
RUMINATECHEW	−5812 ***	−6840 ***	−6680 ***
RUMICHEWBOLUS	−2.52 ***	−5.45 ***	−4.48 ***
RUMIBOUTLENGTH	−6.0 ***	−8.71 ***	−7.2 ***
RUMIBOUTTIME	−73.48 ***	−81.7 ***	−80.1 ***
HACTIVITY	9.89 ***	14.2 ***	10.2 ***
LAYDOWN	−2.0 ***	−1.19 ***	−1.7 ***

P-value: *** < 0.001; ** < 0.01; * < 0.05.

**Table A3:** ANOVA F-test and multiple comparisons tests for the blocks of 60% and 100% herbage allowance groups using each predictor of the combined data.

Variable	S2	S6	M2	M6	L2	L6	F-Test
BITEFREQ	6.2 ***	4.9 ***	2.4 **	4.9 ***	3.5	4.9 ***	***
GRAZINGSTART	−0.13	−0.83	−0.61 ***	−0.65	−1.82	0.60	***
RUMINATECHEW	−2672 **	−8147 ***	−7154 ***	−6517 ***	−11,215 ***	−1674	***
RUMICHEWBOLUS	−0.067	−6.76 ***	−3.12 **	−6.43 ***	−2.85 **	−3.62 ***	***
RUMIBOUTLENGTH	−3.82 **	−8.11 ***	−4.87 ***	−7.65 ***	−10.7 **	−6.27 ***	***
RUMIBOUTTIME	−29.42 *	−93.06 ***	−85.13 ***	−65.85	−154.47 ***	−26.37 ***	***
HACTIVITY	13.44 ***	9.31 ***	7.03	13.08 **	−5.08	25.22 ***	***
LAYDOWN	−2.40 ***	−1.78 ***	−1.13	−1.59 ***	−2.04 ***	−0.92	***

P-value: *** < 0.001; ** < 0.01; * < 0.05.

**Table A4:** Predictive performance of support vector machine based on the estimated sensitivity, specificity, accuracy, positive predictive value (PPV), F-score and the area under receiver operating characteristic curve (AUC) for two-week and six-week restriction period among the cows in early (S), mid (M) and late (L) lactation stage using cross validation studies.

Subset	Sensitivity	Specificity	Accuracy	PPV	F-Score	AUC
S2	0.88 (0.011)	0.82 (0.06)	0.84 (0.005)	0.68 (0.011)	0.76 (0.008)	0.92 (0.005)
S6	0.88 (0.008)	0.90 (0.005)	0.89 (0.004)	0.84 (0.009)	0.86 (0.007)	0.96 (0.002)
M2	0.91 (0.013)	0.82 (0.008)	0.85 (0.006)	0.68 (0.013)	0.78 (0.011)	0.94 (0.006)
M6	0.89 (0.012)	0.83 (0.005)	0.85 (0.005)	0.68 (0.016)	0.76 (0.012)	0.93 (0.004)
L2	0.85 (0.012)	0.85 (0.005)	0.85 (0.004)	0.72 (0.013)	0.77 (0.008)	0.92 (0.004)
L6	0.87 (0.014)	0.79 (0.005)	0.81 (0.004)	0.52 (0.017)	0.64 (0.014)	0.89 (0.005)
W2	0.79 (0.007)	0.83 (0.004)	0.82 (0.003)	0.60 (0.008)	0.68 (0.006)	0.89 (0.003)
W6	0.77 (0.007)	0.87 (0.002)	0.84 (0.002)	0.66 (0.007)	0.70 (0.005)	0.90 (0.003)

Standard errors are indicated in the parentheses.

**Table A5:** Predictive performance of extreme gradient boosting based on the estimated sensitivity, specificity, accuracy, positive predictive value (PPV), F-score and the area under receiver operating characteristic curve (AUC) for two-week and six-week restriction period among the cows in early (S), mid (M) and late (L) lactation stage using cross validation studies.

Subset	Sensitivity	Specificity	Accuracy	PPV	F-Score	AUC
S2	0.89 (0.011)	0.89 (0.007)	0.89 (0.006)	0.83 (0.013)	0.85 (0.009)	0.96 (0.005)
S6	0.89 (0.009)	0.91 (0.005)	0.90 (0.004)	0.85 (0.01)	0.87 (0.006)	0.96 (0.002)
M2	0.85 (0.014)	0.87 (0.008)	0.86 (0.007)	0.79 (0.015)	0.81 (0.011)	0.94 (0.008)
M6	0.85 (0.01)	0.88 (0.004)	0.87 (0.004)	0.79 (0.012)	0.81 (0.009)	0.94 (0.003)
L2	0.85 (0.013)	0.90 (0.006)	0.88 (0.006)	0.81 (0.014)	0.82 (0.011)	0.95 (0.006)
L6	0.90 (0.02)	0.92 (0.003)	0.91 (0.004)	0.83 (0.02)	0.86 (0.015)	0.97 (0.005)
W2	0.72 (0.008)	0.84 (0.004)	0.80 (0.004)	0.64 (0.008)	0.67 (0.006)	0.86 (0.004)
W6	0.75 (0.005)	0.88 (0.003)	0.84 (0.002)	0.71 (0.007)	0.73 (0.004)	0.91 (0.002)

Standard errors are indicated in the parentheses.

**Table A6:** Predictive performance of linear discriminant analysis based on the estimated sensitivity, specificity, accuracy, positive predictive value (PPV), F-score and the area under receiver operating characteristic curve (AUC) for two-week and six-week restriction period among the cows in early (S), mid (M) and late (L) lactation stage using cross validation studies.

Subset	Sensitivity	Specificity	Accuracy	PPV	F-Score	AUC
S2	0.77 (0.003)	0.76 (0.002)	0.76 (0.002)	0.55 (0.003)	0.64 (0.003)	0.84 (0.002)
S6	0.81 (0.002)	0.84 (0.002)	0.83 (0.002)	0.74 (0.003)	0.77 (0.002)	0.90 (0.002)
M2	0.82 (0.003)	0.81 (0.002)	0.81 (0.002)	0.67 (0.003)	0.73 (0.002)	0.89 (0.002)
M6	0.80 (0.003)	0.77 (0.002)	0.78 (0.002)	0.54 (0.003)	0.64 (0.003)	0.86 (0.002)
L2	0.71 (0.003)	0.77 (0.002)	0.75 (0.002)	0.53 (0.004)	0.60 (0.003)	0.81 (0.002)
L6	0.75 (0.003)	0.77 (0.001)	0.76 (0.001)	0.48 (0.003)	0.58 (0.002)	0.82 (0.001)
W2	0.69 (0.003)	0.79 (0.001)	0.77 (0.001)	0.51 (0.003)	0.58 (0.002)	0.81 (0.002)
W6	0.73 (0.003)	0.83 (0.001)	0.81 (0.001)	0.56 (0.003)	0.63 (0.002)	0.85 (0.001)

Standard errors are indicated in the parentheses.

**Table A7:** Predictive performance of neural network based on the estimated sensitivity, specificity, accuracy, positive predictive value (PPV), F-score and the area under receiver operating characteristic curve (AUC) for two-week and six-week restriction period among the cows in early (S), mid (M) and late (L) lactation stage using cross validation studies.

Subset	Sensitivity	Specificity	Accuracy	PPV	F-Score	AUC
S2	0.78 (0.003)	0.77 (0.002)	0.77 (0.002)	0.59 (0.003)	0.66 (0.003)	0.85 (0.002)
S6	0.82 (0.004)	0.86 (0.001)	0.84 (0.002)	0.76 (0.004)	0.79 (0.003)	0.92 (0.002)
M2	0.81 (0.003)	0.82 (0.002)	0.81 (0.002)	0.69 (0.003)	0.74 (0.002)	0.89 (0.002)
M6	0.78 (0.003)	0.78 (0.002)	0.77 (0.002)	0.56 (0.004)	0.64 (0.003)	0.85 (0.002)
L2	0.74 (0.003)	0.80 (0.002)	0.77 (0.002)	0.60 (0.005)	0.65 (0.003)	0.84 (0.002)
W2	0.68 (0.003)	0.80 (0.003)	0.77 (0.001)	0.53 (0.003)	0.59 (0.002)	0.81 (0.003)
W6	0.70 (0.002)	0.83 (0.001)	0.80 (0.001)	0.57 (0.003)	0.62 (0.002)	0.84 (0.002)

Standard errors are indicated in the parentheses.

**Table A8:** Predictive performance of naïve Bayes based on the estimated sensitivity, specificity, accuracy, positive predictive value (PPV), F-score and the area under receiver operating characteristic curve (AUC) for two-week and six-week restriction period among the cows in early (S), mid (M) and late (L) lactation stage using cross validation studies.

Subset	Sensitivity	Specificity	Accuracy	PPV	F-Score	AUC
S2	0.74 (0.003)	0.78 (0.002)	0.76 (0.002)	0.61 (0.003)	0.66 (0.002)	0.83 (0.002)
S6	0.78 (0.003)	0.86 (0.002)	0.82 (0.001)	0.77 (0.003)	0.77 (0.002)	0.90 (0.001)
M2	0.75 (0.003)	0.80 (0.002)	0.77 (0.002)	0.66 (0.003)	0.69 (0.002)	0.85 (0.002)
M6	0.66 (0.003)	0.76 (0.002)	0.72 (0.002)	0.54 (0.003)	0.59 (0.003)	0.78 (0.002)
L2	0.72 (0.003)	0.82 (0.002)	0.79 (0.002)	0.67 (0.004)	0.69 (0.003)	0.86 (0.002)
L6	0.71 (0.003)	0.81 (0.002)	0.78 (0.002)	0.60 (0.004)	0.65 (0.003)	0.84 (0.002)
W2	0.62 (0.003)	0.81 (0.001)	0.75 (0.001)	0.57 (0.003)	0.59 (0.002)	0.80 (0.002)
W6	0.64 (0.003)	0.85 (0.001)	0.79 (0.001)	0.64 (0.003)	0.63 (0.002)	0.83 (0.002)

Standard errors are indicated in the parentheses.

## References

[1] Zehner N., Umstätter C., Niederhauser J.J., Schick M. (2017). System specification and validation of a noseband pressure sensor for measurement of ruminating and eating behavior in stable-fed cows. Comput. Electron. Agric..

[2] Alsaaod M., Niederhauser J.J., Beer G., Zehner N., Schuepbach-Regula G., Steiner A. (2015). Development and validation of a novel pedometer algorithm to quantify extended characteristics of the locomotor behaviour of dairy cows. J. Dairy Sci..

[3] Greenwood P.L., Paull D.R., McNally J., Kalinowski T., Ebert D., Little B., Smith D.V., Rahman A., Valencia P., Ingham A.B. (2018). Use of sensor-determined behaviours to develop algorithms for pasture intake by individual grazing cattle. Crop Pasture Sci..

[4] Rombach M., Münger A., Niederhauser J., Südekum K.H., Schori F. (2018). Evaluation and validation of an automatic jaw movement recorder (RumiWatch) for ingestive and rumination behaviors of dairy cows during grazing and supplementation. J. Dairy Sci..

[5] Rombach M., Südekum K.H., Münger A., Schori F. (2019). Herbage dry matter intake estimation of grazing dairy cows based on animal, behavioral, environmental, and feed variables. J. Dairy Sci..

[6] Werner J., Umstatter C., Kennedy E., Grant J., Leso L., Geoghegan A., Shaloo L., Schick M., O’Brian B. (2019). Identification of possible cow grazing behaviour indicators for restricted grass availability in a pasture-based spring calving dairy system. Livestock Sci..

[7] R Core Team (2019). R: A language and environment for statistical computing. R Foundation for Statistical Computing, Vienna, Austria.

[8] Rahman A., Smith D.V., Little B., Ingham A.B., Greenwood P.L., Bishop-Hurley G.J. (2018). Cattle behaviour classification from collar, halter, and ear tag sensors. Inf. Process. Agric..

[9] Werner J., Leso L., Umstatter C., Niederhauser J., Kennedy E., Geoghegan A., Shalloo L., Schick M., O’Brien B. (2018). Evaluation of the RumiWatchSystem for measuring grazing behaviour of cows. J. Neurosci. Methods.

[10] Kuhn M. (2008). Building predictive models in R using the caret package. J. Stat. Softw..

[11] Venables W.N., Ripley B.D. (2002). R package class. Modern Applied Statistics with S.

[12] Karatzoglou A., Meyer D., Hornik K. (2006). Support Vector Machines in R. J. Stat. Softw..

[13] Therneau T., Atkinson B., Ripley B., Ripley M.B. (2019). rpart: Recursive Partitioning and Regression Trees. R Package Version 4.1–15.

[14] Liaw A., Wiener M. (2002). Classification and Regression by randomForest. R News.

[15] Chen T., He T., Benesty M., Khotilovich V., Tang Y., Cho H., Chen K., Mitchell R., Cano I., Zhou T. (2019). xgboost: Xgboost: Extreme gradient boosting. R package Version 0.90.0.2.

[16] Fielding A.H., Bell J.F. (1997). A review of methods for the assessment of prediction errors in conservation presence/ absence models. Environ. Conserv..

[17] Steensels M., Antler A., Bahr C., Berckmans D., Maltz E., Halachmi I. (2016). A decision-tree model to detect post-calving diseases based on rumination, activity, milk yield, BW and voluntary visits to the milking robot. Animal.

[18] Friedman J.H. (2001). Greedy function approximation: A gradient boosting machine. Ann. Stat..

[19] Heise H., Theuvsen L. (2018). Citizens’ understanding of welfare of animals on the farm: An empirical study. J. Appl. Anim. Welfare Sci..

[20] FAO (2017). The Future of Food and Agriculture—Trends and Challenges.

